# Clinical features and 28-day mortality predictors of vaccinated patients admitted to a COVID-19 ICU hub in Italy

**DOI:** 10.1186/s44158-023-00130-6

**Published:** 2023-11-13

**Authors:** Claudia Stella, Cecilia Berardi, Annalisa Chiarito, Veronica Gennenzi, Stefania Postorino, Donatella Settanni, Melania Cesarano, Rikardo Xhemalaj, Eloisa Sofia Tanzarella, Salvatore Lucio Cutuli, Domenico Luca Grieco, Giorgio Conti, Massimo Antonelli, Gennaro De Pascale

**Affiliations:** 1 Dipartimento Di Scienze Biotecnologiche Di Base, Cliniche Intensivologiche E Perioperatorie, Università Cattolica del Sacro Cuore, Rome, Italy; 2 Dipartimento Di Scienze Dell’Emergenza, Anestesiologiche E Della Rianimazione, Fondazione Policlinico Universitario A. Gemelli IRCCS, Rome, Italy

**Keywords:** COVID-19, Vaccination, Mortality, Intensive care unit

## Abstract

**Background:**

COVID-19 vaccination has been proved to be effective in preventing hospitalization and illness progression, even though data on mortality of vaccinated patients in the intensive care unit (ICU) are conflicting. The aim of this study was to investigate the characteristics of vaccinated patients admitted to ICU according to their immunization cycle and to outline the risk factors for 28-day mortality. This observational study included adult patients admitted to ICU for acute respiratory failure (ARF) due to SARS-CoV-2 and who had received at least one dose of vaccine.

**Results:**

Fully vaccination was defined as a complete primary cycle from < 120 days or a booster dose from > 14 days. All the other patients were named partially vaccinated. One-hundred sixty patients (91 fully and 69 partially vaccinated) resulted eligible, showing a 28-day mortality rate of 51.9%. Compared to partially vaccinated, fully vaccinated were younger (69 [60–77.5] vs. 74 [66–79] years, *p* 0.029), more frequently immunocompromised (39.56% vs. 14.39%, *p* 0.003), and affected by at least one comorbidity (90.11% vs 78.26%, *p* 0.045), mainly chronic kidney disease (CKD) (36.26% vs 20.29%, *p* 0.035). At multivariable analysis, independent predictors of 28-day mortality were as follows: older age [*OR* 1.05 (*CI* 95% 1.01–1.08), *p* 0.005], history of chronic obstructive pulmonary disease (COPD) [*OR* 3.05 (*CI* 95% 1.28–7.30), *p* 0.012], immunosuppression [*OR* 3.70 (*CI* 95% 1.63–8.40), *p* 0.002], and admission respiratory and hemodynamic status [PaO_2_/FiO_2_ and septic shock: *OR* 0.99 (*CI* 95% 0.98–0.99), *p* 0.009 and 2.74 (*CI* 95% 1.16–6.48), *p* 0.022, respectively].

**Conclusions:**

Despite a full vaccination cycle, severe COVID-19 may occur in patients with relevant comorbidities, especially immunosuppression and CKD. Regardless the immunization status, predisposing conditions (i.e., older age, COPD, and immunosuppression) and a severe clinical presentation were predictors of 28-day mortality.

## Background

The coronavirus disease (COVID-19) spread in Europe in February 2020, and it was associated with almost 200,000 deaths in Italy [1]. In Italy, the vaccination campaign started in December 2020 [2], and BNT162b2 (Pfizer-BioNTech), mRNA-1273 (Moderna), ChAdOx1 (AstraZeneca), and Ad26.COV2.S (Janssen) were adopted for immunization. With the exception of Janssen that was administered in a single dose, the primary vaccination cycle consisted in two doses injected at a distance of 3–12 weeks, depending on the vaccine. From September 2021, a “booster” dose was offered to high-risk patients, and from November 2021, it was extended to the entire adult population [3]. Worldwide, the vaccination resulted effective in protecting from hospitalization and illness progression [4–6]. Indeed, it was reported that unvaccinated patients accounted for more than 80% of COVID-19 admissions [4], while hospitalization for COVID-19, 28-day mortality, and invasive mechanical ventilation was significantly associated with decreased likelihood of vaccination [4]. The prevention of hospital admission was then confirmed regardless the variant of SARS-CoV-2, although a “booster” dose was necessary to achieve a protection from Omicron comparable to Alpha and Delta variants [5]. However, it was also demonstrated that immunization tended to wane over time: a reduction of vaccines effectiveness against SARS-CoV-2 infection is shown after 120 days from the last administration [7], and patients at risk (≥ 65 years) are reported to have a significant drop below 50% of protection for symptomatic COVID-19 after 15 weeks from the immunization with Moderna (while it remained > 50% with Pfizer) [8]. On the other hand, effectiveness against hospitalization and mortality remained sustained even after 20 weeks from vaccination [8], and data from a large meta-regression indicated an overall preserved protection against severe COVID-19 for up to 6 months (effectiveness > 50%) [9]. Among patients admitted to intensive care unit (ICU), vaccinated were characterized by older age [10, 11] and a higher number of comorbidities [10, 12]. In particular, what emerged from different studies is that vaccinated patients were more often affected by chronic heart disease (CHD), diabetes mellitus (DM), chronic kidney disease (CKD), chronic obstructive pulmonary disease (COPD), and conditions which determine immunosuppression [13–15]. In terms of outcomes, mortality in ICU was reported to be either similar [12, 13, 16], decreased [15], or increased [10] when vaccinated patients were compared to non-vaccinated. For what concerns the risk factors for mortality, in the pre-vaccination era, they essentially overlapped with the conditions predisposing to ICU admission [17–19], while fewer studies were focused on populations of only vaccinated patients [20].

Thus, the aim of this study was to describe the main characteristics of vaccinated patients according to the time from immunization and to identify the predictors of 28-day mortality in a hub ICU during the pandemic breakthrough.

## Methods and materials

### Study setting and design

This observational prospective study was conducted in the ICU of “Fondazione Policlinico Universitario A. Gemelli IRCCS,” which acted as hub ICU for the entire Lazio region during the COVID-19 breakthrough. Patients were consecutively enrolled from June 2021 to May 2022. The study was performed in accordance with the Declaration of Helsinki and was approved by the ethics committee (FPG-UCSC reference number ID3141). A written informed consent or proxy consent was waived, due to observational nature of the study, according to committee recommendations. All data were anonymous and identified with an admission code number. Adult patients (> 18 years) with an active infection from SARS-CoV-2, with a known vaccination status, and admitted to ICU for acute respiratory failure (ARF) were eligible. Active infection was defined as positive results of either nasal pharyngeal swab or distal samples, such as tracheobronchial secretions or bronchoalveolar lavage, before ICU admission. Exclusion criteria were as follows: non-vaccinated patients, unavailable data on vaccination status, admission for other causes beside ARF, and patients lost in follow-up. We reported data on demographics (age, sex) and relevant comorbidities, i.e., obesity (body mass index—BMI > 30 kg/m^2^), chronic heart failure (CHF), chronic obstructive pulmonary disease (COPD), chronic kidney disease (CKD), and diabetes. Immunocompromission, as a risk factor for COVID-19, was defined according to CDC directive [21]. Invasive mechanical ventilation (IMV), ratio of arterial pressure of oxygen to inspired fraction of oxygen (PaO_2_/FiO_2_), and pharmacological treatments at admission were recorded, as well as the development of complications by 48 h from the beginning of the ICU stay, including septic shock, acute kidney injury (AKI) requiring continuous renal replacement therapy (CRRT), barotrauma (pneumothorax or pneumomediastinum), venous thromboembolism (VTE, including pulmonary embolism), and concomitant infection. Electronic patient records (Digistat®) and microbiology laboratory data (TrakCare®) were used to identify patients and to retrieve clinical and microbiological results. Subjects who received either two doses of BNT162b2 (Pfizer-BioNTech), mRNA-1273 (Moderna), and ChAdOx1 (AstraZeneca), or one dose of Ad26.COV2.S (Janssen) from less than 120 days, or a third “booster” dose from more than 14 days were defined as “fully vaccinated.” Patients who were given any other number of doses or in a different interval of time were named “partially vaccinated.” Primary outcome of the study was to outline the clinical features of fully vaccinated patients compared to partially vaccinated, 28-day mortality, and its predictors. Secondary outcomes were inhospital mortality, the development of ICU-acquired infections, and barotrauma.

### Statistical analysis

The Kolmogorov–Smirnov test was used to evaluate the distribution of variables. Data with a non-normal distribution were assessed with the Mann–Whitney test, and the median and selected centile (25th–75th) values are given. The data with a normal distribution were assessed with the Student’s *t*-test. Categorical variables are given as proportions and were analyzed with the chi-square test or Fisher’s exact test, as appropriate. *p* < 0.05 was considered significant. The crude odds ratio (OR) and 95% CI were calculated for each variable. We included all variables in the multivariable logistic regression if they reached *p* ≤ 0.1 on univariate analysis. A stepwise selection procedure was used to select variables for inclusion in the final model. Overall goodness of fit was analyzed by Nagelkerke’s R-square. All statistical analyses were performed using SPSS Statistical Software version 28.0.1.0 (IBM Corporation, Armonk, NY, USA), whereas data were graphed using GraphPad Prism version 6.0 (GraphPad Software, San Diego, CA, USA).

### Microbiological analysis

Nasopharyngeal swabs were obtained from COVID-19 patients to detect one or more SARS-CoV-2-specific nucleic acid targets by the Korean Ministry of Food and Drug Safety approved Allplex™ 2019-nCoV assay (Arrow Diagnostics S.r.l., Genova, Italy), which is a real-time reverse-transcriptase–polymerase-chain-reaction (RT-PCR)-based assay for SARS-CoV-2 RNA detection. A positive RT-PCR result was used to confirm COVID-19 diagnosis, which in turn relied on the presence of fever and/or lower-respiratory-tract symptoms and on lung imaging features consistent with SARS-CoV-2 pneumonia.

For the diagnosis of bacterial concomitant pneumonia, the respiratory samples were immediately sent to the microbiology laboratory for microbiological investigations, consisting of Gram staining examination and (qualitative or quantitative) aerobic cultures on standard agar media. For microbial isolates, species identification was performed using the MALDI Biotyper system (Bruker Daltonics, Bremen, Germany), and in vitro antimicrobial susceptibility testing was performed using Vitek 2 (bioMérieux, Mercy l’Étoile, France) or MERLIN Diagnostica GmbH (Bornheim, Germany) broth microdilution systems. Minimum inhibitory concentrations were interpreted in accordance with the European Committee on Antimicrobial Susceptibility Testing (EUCAST) clinical breakpoints.

## Results

**Fig. 1:**
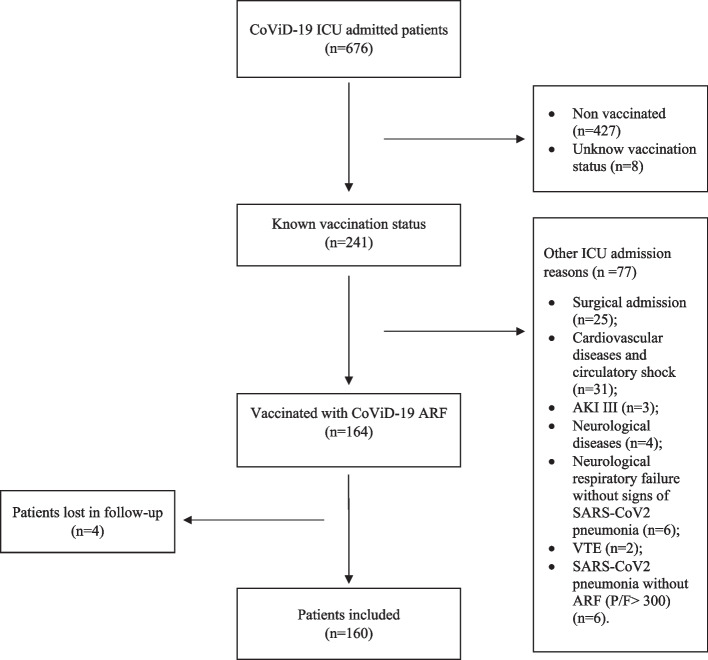
Enrollment process flowchart. AKI, acute kidney injury; ARF, acute respiratory failure; P/F, PaO2 to FiO2 ratio; VTE, venous thromboembolism

During the study period, a total of 676 patients with an active infection from SARS-CoV-2 were admitted to ICU. Vaccinated patients were 241 (35.65%), 160 of whom fulfilled the inclusion criteria and resulted eligible for the final analysis. The enrollment process is represented in Fig. 1. Demographics, comorbidities, presenting features, and ongoing treatment at ICU admission are summarized in Table 1.

**Table 1:** General characteristics patients classified according to vaccination status

Variables	Total cohort (*n* = 160)	Partially vaccinated (*n* = 69)	Fully vaccinated (*n* = 91)	*p*-value
***Demographics and comorbidities***				
Age, years	71 (61.8–78)	74 (66–79)	69 (60–77.5)	.029*
Gender (male)	111 (69.38%)	44 (63.77%)	67 (73.63%)	0.226
The presence of at least one comorbidity	136 (85.00%)	54 (78.26%)	82 (90.11%)	.045*
BMI ≥ 30 kg/m^2^	24 (15.00%)	11 (15.94%)	13 (14.29%)	0.825
CHD	53 (33.13%)	19 (27.54%)	34 (37.36%)	0.236
COPD	40 (25.00%)	24 (34.78%)	16 (17.58%)	.016*
Diabetes	47 (29.38%)	24 (34.78%)	23 (25.27%)	0.222
CKD	47 (29.38%)	14 (20.29%)	33 (36.26%)	.035*
Immunosuppression	48 (30.00%)	12 (14.39%)	36 (39.56%)	.003*
***Clinical ICU presenteng features***				
Pre-ICU hospital LOS, days	3 (1–8)	3 (1–5)	4 (1–12)	.04*
PaO_2_/FiO_2_	109 (83–146.5)	100 (81.5–144)	114 (83.5–152)	0.364
Ongoing IMV	50 (31.25%)	20 (28.99%)	30 (32.97%)	0.610
Septic shock	44 (27.50%)	20 (28.99%)	24 (26.37%)	0.724
AKI III requiring CRRT	26 (16.25%)	8 (11.60%)	18 (19.78%)	0.198
Barotrauma	16 (10.00%)	6 (8.70%)	10 (10.99%)	0.792
Pulmonary embolism	12 (7.50%)	4 (5.80%)	8 (8.79%)	0.556
Concomitant infection	58 (36.25%)	22 (31.88%)	36 (39.56%)	0.407
***Treatments at ICU admission***				
Dexamethasone	135 (84.38%)	66 (95.65%)	69 (75.82%)	< .0001*
Remdesivir	71 (44.38%)	36 (52.17%)	35 (38.46%)	0.108
IL-6 inhibitors	31 (19.38%)	14 (20.29%)	17 (18.68%)	0.842
Insulin	61 (38.13%)	23 (33.33%)	38 (41.76%)	0.325
Antibiotics	102 (63.75%)	38 (55.07%)	64 (70.33%)	.067

Categorical variables are expressed in count and percentage; continuous variables are expressed in median and interquartile range. *p* < 0.05 was considered significant and marked with “asterisk” (*)

*AKI*, acute kidney injury; *BMI*, body mass index; *CHD*, chronic heart disease; *COPD*, chronic obstructive pulmonary disease; *CKD*, chronic kidney disease; *HFO*, high-flow oxygen; *ICU*, intensive care unit; *IL-6*, interleukin-6; *IMV*, invasive mechanical ventilation; *NIV*, noninvasive ventilation; *LOS*, length of stay; *SAPS II*, Simplified Acute Physiology Score II; *SOFA*, Sequential Organ Failure Assessment

### Vaccination status and clinical features

**Fig. 2:**
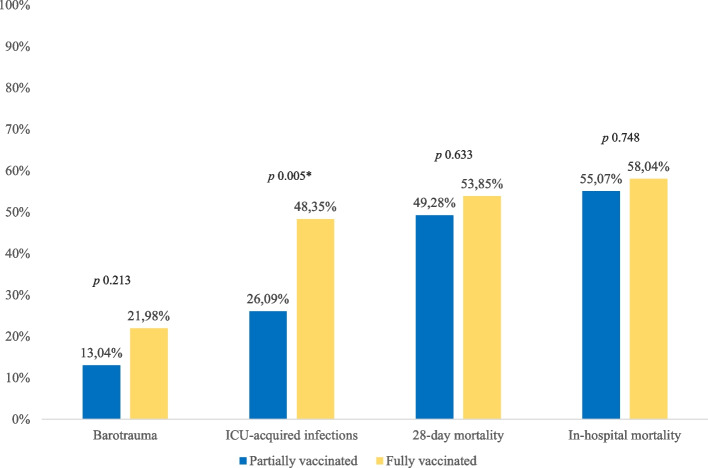
Clinical outcomes according to vaccination status *p* <0.05 was considered significant and marked with “asterisk” (*)

The comparison of clinical characteristics of fully and partially vaccinated patients is shown in Table 1. Fully vaccinated accounted for 56.87% (*n* = 91) of the patients, and they resulted to be younger (69 [60–77.5] vs. 74 [66–79] years, *p* 0.029, respectively). Moreover, they were more frequently affected from at least one comorbidity (90.11% vs 78.26%, *p* 0.045). In detail, with the exception of COPD which had a higher prevalence among in the partially vaccinated population (17.58% vs 34.78%, *p* 0.016), fully vaccinated patients suffered more from CKD (36.26% vs 20.29%, *p* 0.035) and immunesuppression (39.56% vs 14.39%, *p* 0.003). Concerning clinical outcomes (Fig. 2), there were no differences in terms of complication with barotrauma, inhospital mortality, and 28-day mortality between fully and partially vaccinated. On the other hand, fully vaccinated developed more frequently ICU-acquired infections (48.35% vs 26.09%, *p* 0.005).

### Predictors of 28-day mortality

Eighty-three patients out of 160 did not survive at 28 days, showing a mortality rate of 51.86%. Results of univariate and multivariate logistic regressions are shown in Table 2. At univariate analysis, among the demographic and anamnestic features, predisposing factors to 28-day mortality were older age [*OR* 1.04 (95% *CI* 1.01–1.07), *p* 0.005], the presence of at least one comorbidity [*OR* 3.08 (95% *CI* 1.20–7.90), *p* 0.02], COPD [*OR* 2.37 (95% *CI* 1.12–5.04), *p* 0.024], and immunosuppression [*OR* 2.73 (95% *CI* 1.33–5.58), *p* 0.006]. Instead, clinical predictors were PaO_2_/FiO_2_ ratio [*OR* 0.99 (95% *CI* 0.98–0.99), *p* 0.002] and the development of septic shock by 48 h after ICU admission [*OR* 3.40 (95% *CI* 1.59–7.25), *p* 0.002]. Notably, a complete vaccination cycle [*OR* 1.20 (95% *CI* 0.64–2.25), *p* 0.567] and the administration of a booster dose [*OR* 1.52 (95% *CI* 0.80–2.89), *p* 0.206] were not correlated with the risk of death by day 28. When studied together in a multivariate logistic regression model, factors that maintained a significant association with 28-day mortality were age [*OR* 1.05 (95% *CI* 1.01–1.06), *p* 0.005], history of COPD [*OR* 3.05 (95% *CI* 1.28–7.30), *p* 0.012], immunosuppression [*OR* 3.70 (95% *CI* 1.63–8.40), *p* 0.002], PaO_2_/FiO_2_ ratio at admission [*OR* 0.99 (95% *CI* 0.98–0.99), *p* 0.009], and septic shock in the first 48 h [*OR* 2.74 (95% *CI* 1.16–6.48), *p* 0.022].

**Table 2:** Univariate and multivariate analysis of factors associated with 28-day mortality

			**Univariate**		**Multivariate**	
**Variables**	**Survivors (*n* = 77)**	**Nonsurvivors (*n* = 83)**	***p*-value**	**OR (95% CI)**	***p*-value**	**OR (95% *CI*)**
***Demographics and comorbidities***						
Age, years	67 (59.5–75)	75 (66–79)	.005*	1.04 (1.01–1.07)	.005*	1.05 (1.01–1.08)
Gender (male)	59 (71.08%)	52 (62.65%)	.057	0.51 (0.26–1.02)	0.400	0.71 (0.32–1.58)
Fully vaccinated	42 (50.60%)	49 (59.03%)	0.567	1.20 (0.64–2.25)		
Vaccination booster dose	25 (32.47%)	35 (42.17%)	0.206	1.52 (0.80–2.89)		
The presence of at least one comorbidity	60 (72.29%)	76 (91.57%)	.02*	3.08 (1.20–7.90)		
BMI ≥ 30 kg/m^2^	11 (13.25%)	13 (15.66%)	0.808	1.11 (0.47–2.66)		
CHD	24 (28.92%)	29 (34.94%)	0.613	1.19 (0.61–2.30)		
COPD	13 (15.66%)	27 (32.53%)	.024*	2.37 (1.12–5.04)	.012*	3.05 (1.28–7.30)
Diabetes	24 (28.92%)	23 (27.71%)	0.632	0.85 (0.43–1.67)		
CKD	19 (22.89%)	28 (33.73%)	0.210	1.55 (0.78–3.10)		
Immunosuppression	15 (18.07%)	33 (39.76%)	.006*	2.73 (1.33–5.58)	.002*	3.70 (1.63–8.40)
Charlson’s comorbidity index	5 (3–7)	6 (5–7)	.081	1.12 (0.99–1.26)		
***Clinical ICU presenting features***						
Pre-ICU hospital LOS, days	3 (1–8)	4 (1–10)	0.253	1.02 (0.98–1.06)		
PaO_2_/FiO_2_	122 (92–175)	100 (71–133)	.002*	0.99 (0.98–0.99)	.009*	0.99 (0.98–0.99)
Ongoing IMV	23 (27.71%)	27 (32.53%)	0.717	1.13 (0.58–2.21)		
Septic shock	12 (14.46%)	32 (38.55%)	.002*	3.40 (1.59–7.25)	.022*	2.74 (1.16–6.48)
AKI III requiring CRRT	14 (16.87%)	12 (14.46%)	0.524	0.76 (0.33–1.77)		
Barotrauma	6 (7.23%)	10 (12.05%)	0.373	1.62 (0.56–4.70)		
Venous thromboembolism	4 (4.82%)	8 (9.64%)	0.293	1.95 (0.56–6.75)		
Concomitant infection	23 (27.71%)	35 (42.17%)	0.107	1.71 (0.89–3.29)		
***Treatments at ICU admission***						
Remdesivir	40 (49.19%)	31 (37.35%)	.064	0.55 (0.29–1.04)	0.220	0.622 (0.29–1.33)
Dexamethasone	67 (80.72%)	68 (81.93%)	0.378	0.68 (0.28–1.61)		
IL-6 inhibitors	14 (16.87%)	17 (20.48%)	0.713	1.16 (0.53–2.55)		
Insulin	28 (33.73%)	33 (39.76%)	0.659	1.16 (0.61–2.19)		
Antibiotics	46 (55.42%)	56 (67.47%)	0.310	1.40 (0.73–2.67)		
***Outcomes***						
Barotrauma	12 (14.46%)	17 (20.48%)	0.423	1.40 (0.62–3.15)		
ICU-acquired infections	26 (31.33%)	36 (43.37%)	0.214	1.50 (0.79–2.85)		

*p *<0.05 was considered significant and marked with “asterisk” (*)*. AKI*, acute kidney injury; *BMI*, body mass index; *CHD*, chronic heart disease; *COPD*, chronic obstructive pulmonary disease; *CKD*, chronic kidney disease; *HFO*, high-flow oxygen; *ICU*, intensive care unit; *IL-6*, interleukin-6; *IMV*, invasive mechanical ventilation; *NIV*, noninvasive ventilation; *LOS*, length of stay; *SAPS II*, Simplified Acute Physiology Score II; *SOFA*, Sequential Organ Failure Assessment

## Discussion

COVID-19 has been associated with hundreds of thousands of deaths in Italy over 3 years [1]. The main measure adopted to contrast it was the introduction of vaccines, which resulted effective in controlling the viral diffusion [22–24] and its most severe consequences [4–6, 10, 12]. In our population, 35.65% of patients admitted to ICU had received at least one dose of vaccine, which was aligned with data from other Italian ICUs (26–43%) [10, 12, 15]. Similar to what previously reported [10, 13, 17], it emerged from our cohort that vaccinated patients were in most of the cases high-risk patients, i.e., age > 65 years (71 [61.8–78] years) and affected by at least one of the comorbidities that were demonstrated to be associated to worse clinical outcomes (85% patients) [13–15, 25], such as obesity, CHD, COPD, diabetes, CKD, and immunosuppression.

We investigated fully and partially vaccinated patients separately in order to identify differences at admission and higher level of protection in terms of clinical outcomes. In fact, both the time intercurred from the last administration [7–9] and the number of doses [5, 6, 26, 27] were reported to significantly influence the level of immunization from COVID-19, likely due to the waning of antibody titer and the emergence of new variants during the pandemic breakthrough [26, 28]. Indeed, vaccine effectiveness was shown to start decreasing after 120 days [7], and the administration of the last dose from < 120 days was associated with the lower incident rate ratio for ICU admission (*IRR* 0.03, 95% *CI* 0.03–0.04) [12]. In parallel, a third booster dose was more effective against all variants, included Omicron [5, 26, 28, 29], and in patients at higher risk, such as immunocompromised [27, 30, 31].

When split according to the immunization cycle, fully vaccinated resulted to be younger than partially vaccinated yet—not surprisingly—more often had comorbidities, specifically CKD and immunocompromission. For what concerns immunocompromising conditions, they were already reported as associated with reduced effectiveness of vaccination [4], demonstrated by a tempered seroconversion especially among organ transplant recipients [32, 33], patients with hematological cancers [34], immune-mediated inflammatory disorders, and solid cancers [35]. Moreover, the scarce seroconversion was shown to be correlated to a reduced clinical efficacy of the vaccine in preventing hospitalization and death [36], despite an improvement of antibody response proportional to the number of doses received [27, 31, 35]. This was not confirmed in our population, where the immunocompromised patients were more often fully vaccinated; thus, a higher number of doses or a shorter administration interval did not show the protective effect against ICU admission. On the other hand, an important bias could be the national recommendations for the booster dose, as subjects at risk were prioritized compared to the general population. Therefore, proportionally more immunocompromised people were likely to have received a third dose before the rest of the population [3]. Interestingly, in spite of the disparity in the distribution of comorbidities, the presenting features and the early complications were comparable between the two groups (Table 1).

In terms of clinical outcomes, immunosuppression status and the longer hospitalization before ICU (3 [1–5] vs 4 [1–12] days, *p* 0.04, for partially and fully vaccinated, respectively) could give reason of the higher incidence of ICU-acquired infections detected among fully vaccinated patients. By the way, in our cohort, ICU-acquired infections did not affect mortality at univariate analysis [*OR* 1.50 (95% *CI* 0.79–2.85), *p* 0.214]. On the other hand, clinical outcomes, such as the development of barotrauma and inhospital and 28-day mortality, did not differ significantly, suggesting vaccine effectiveness lasting for more than 120 days. Finally, the protective effect of vaccination from mortality regardless the number of doses and the time intercurred was confirmed by the univariate analysis, which showed that a “fully vaccinated” status and the previous administration of a booster dose were not associated with reduced risk of death [*OR* 1.20 (95% *CI* 0.64–2.25), *p* 0.567 and *OR* 1.52 (95% *CI* 0.80–2.89), *p* 0.206, respectively].

We found a 28-day mortality rate of 51.86%, which is comparable to what reported in other studies from different countries where mortality of vaccinated patients admitted to ICU ranged between 24.3 and 58% [10, 12–15, 20]. In our population, the comorbidities independently associated with 28-day mortality were older age, COPD, and immunosuppression.

These results are consistent with what already known from the COVID-19 experience. In fact, age was identified as the main risk factor for mortality both in the general population, with people older than 65 years accounting for 75–81% of deaths for COVID-19 [37, 38], and among the critically ill patients [12, 17, 18, 25, 39]. The increased susceptibility of the elderly to viral infections, respiratory failure, and death are likely to be linked to the physiological changes that occur with aging, such as immunosenescence (i.e., the age-related immune dysregulation) [37]. As an example, it was demonstrated that people ≥ 60 years are overrepresented among low antibody responders to COVID-19 vaccines [29], even though it did not reach a clinical significance in terms of reduced effectiveness of vaccination against the development of severe forms and hospitalization [4, 29]. Thus, other alterations could give reason of the higher mortality, such as the decreased respiratory capacity, greater incidence of infections and sepsis, and frailty [37].

COPD was previously associated with increased risk of severe COVID-19, ICU admission, and mortality [40–42]. Different mechanisms are proposed to lay under the increased risk, i.e., the reduced basal respiratory function, the higher incidence of active smoking and its consequent systemic inflammatory state which establishes a favorable environment for severe SARS-CoV-2 infection [40], and the upregulated expression of angiotensin-converting enzyme 2 (ACE2) receptors, the same receptor used by SARS-CoV-2 to infect host cells [40–42]. In our population, the predisposing role of COPD to 28-day mortality was coherent with the existent literature.

Consistently with the data present in literature, another predisposing factor to 28-day mortality outlined by our analysis was immunocompromission. In fact, in addition to the abovementioned reduced vaccine effectiveness in these cohort, immunocompromised patients have three to four times higher mortality rates compared to non-immunosuppressed subjects [43, 44].

For what concerns the clinical presenting features, lower PaO_2_/FiO_2_ ratio and the development of septic shock by 48 h from ICU admission independently predisposed to 28-day mortality in our cohort. The former was already associated with increased mortality [12, 17, 18, 20], presumably as an expression of the severity of the respiratory failure [45], while the latter is well-known to be burdened with high mortality rate [46], and it was already listed as one of the main causes of death among COVID-19 patients [39, 47, 48]. Furthermore, patients with a concomitant infection from SARS-CoV-2 are at higher risk of dying because of sepsis and septic shock when compared to non-COVID-19 population [49]. Thus, the data we obtained from our analysis are consistent with what previously reported in literature.

The study presented hereby has some limitations. Firstly, the single-center design of the study intrinsically reduces the extendibility of results, as they might have been influenced by the local clinical practice. In spite of this, our findings are comparable to those obtained by other authors in different centers. Secondly, data on the antibody titer and, thus, the actual seroconversion after vaccination were not available. Additionally, clinical outcomes were not stratified according to the type of vaccine received (i.e., mRNA versus viral vector technologies). Finally, we analyzed mortality and its predictors only for the first 28 days after ICU admission because longer-term data were not available for the entire cohort. Indeed, after 1 month, many patients were discharged from our institution (i.e., discharged home, transferred to other hospitals, transferred to long-term facilities), so we did not have access to the records of all patients.

## Conclusions

Despite a full vaccination cycle, severe COVID-19 may occur in patients with relevant comorbidities, especially immunosuppression and CKD. Regardless the immunization status, predisposing conditions (i.e., older age, COPD, and immunosuppression) and a worse clinical presentation were predictors of 28-day mortality.

## Data Availability

The datasets used and/or analyzed during the current study are available from the corresponding author on reasonable request.
